# Bioactive Compounds Produced in Leaves of Mulberry (*Morus alba* L.) Transplants under Modified Environments of Root and Aerial Zones

**DOI:** 10.3390/plants11212850

**Published:** 2022-10-26

**Authors:** Aye Nwe Win, Darunmas Sankhuan, Watcharra Chintakovid, Kanyaratt Supaibulwatana

**Affiliations:** 1 Department of Biotechnology, Faculty of Science, Mahidol University, Bangkok 10400, Thailand; 2 Sericulture Research and Development Center, Pyin-Oo-Lwin, Mandalay 05081, Myanmar; 3 Agricultural Science Program, Kanchanaburi Campus, Mahidol University, Kanchanaburi 71150, Thailand

**Keywords:** mulberry, transplants, soilless cultures, LED, light spectrum, bioactive compound

## Abstract

Different shoot/root micro-environments were investigated for growth performances and nutraceutical compounds in leaves of mulberry (*Morus alba* L.) transplants. Single-node segments were taken from seedling-grown pots of three cultivars: Myanmar large leaf (MLL), Myanmar medium leaf (MML), and C14. Transplant production was compared in soil, vermiculite (V), or the dynamic root floating technique (DRFT). The highest survival percentage of the transplants was obtained from V-system, and MLL showed a higher shoot/root formation over two tested cultivars. The MLL transplants grown in V-system under white LED light (445 and 554 nm) at 200 μmol·m^−2^·s^−1^ gave a fresh weight with superior qualified transplants compared to other treatments. The bioactive compounds in leaves of MLL, MML, and C14 were analyzed using GC–MS after incubation with different LED spectra. Ethanol extracts of the leaves revealed that more than 50% of the bioactive compounds were fatty acids and conjugates and varied according to spectra and cultivar. Blue LED light (445 nm) induced the production of total phenolics, whereas white LED light favored the production of total proteins, soluble sugar, and biomass. The modified environments at the root and aerial zones significantly influenced the growth and biochemical parameters of transplants, and this applied technique can elevate useful functional ingredients of mulberry leaves.

## 1. Introduction

Mulberry (*Morus alba* L.), a perennial woody tree, is well-known for its rapid growth and biomass production [1,2]. In sericulture, mulberry leaves play an essential role as the sole food source for silkworm (*Bombyx mori*) rearing. Mulberry cultivation provides a large number of mulberry leaves for sericulture industry in many countries. Mulberry leaves are widely used for their pharmaceutical value as traditional herbs because of their chemical constituents [3,4], and also used as a raw material for herbal tea. Mulberry plantations are promoted by the Myanmar Government to support the local silk hand-weaving industry, especially in areas such as Kayin State where primary crops such as paddy (*Oryza sativa*) and rubber (*Hevea brasiliensis*) have low market value and fail to sustain local livelihoods.

Single-node (or single eye) cuttings are one of the methods used for mulberry production when space or stock material is limited. Due to the high heterozygosity and long juvenile period, vegetative propagation methods such as stem cutting and grafting have become the preferred choices for commercial mulberry cultivation [5,6]. Conventional methods of mulberry cutting require more space and time. Cutting at the initial growth level (with at least 4–5 nodes) requires 18–20 months of propagation and less-than-conventional cutting before supplying to growers. However, the proper stage of stock plants can be delayed by drought or climate change and these limit the contribution of stock plants to mulberry growers.

Balancing the high demands of medicine for health issues and the supply of natural products obtained from limited natural resources is a challenge. Establishing an alternative technology to produce nutraceutical substances on an industrial scale would be hugely beneficial. Nutraceuticals are products isolated from herbs, dietary supplements, specific diets, and processed foods that are also used as medicine [7]. Recently, using biological organisms or plants as manufacturing units to produce functional or nutraceutical substances has increased [8]. Plant factory systems have been implemented to increase the productivity of bioactive compounds in medicinal plants. Plant factories use modern technology to grow plants in a controlled environment, enabling year-round and precise production of vegetables and some species of annual herbs [9]. It is possible to manipulate micro-environments such as light, temperature, water, and nutrients to control growth and nutraceutical substances in leafy vegetables; however, the growth of perennial trees or stock plants has rarely been reported in plant factories.

Light environments (light quantity, light quality, and light duration) play important roles in plant growth and development, as well as promoting biosynthesis of bioactive compounds in vegetables and sprouts [10,11,12,13,14,15,16,17,18,19,20]. LEDs (light emitting diodes) offer many advantages over other light sources such as wavelength specificity, the narrow light spectrum, and low heat production [21]. The broad wavelength in white LED light is composed of blue, green, and red light. White LED light has a more specific spectral distribution than conventional white fluorescent lamps for commercial plant cultivation [22]. However, inadequate information is available to select the appropriate light quality for growing perennial woody trees (such as mulberry) or mini-scale cuttings of woody plants.

The objective of this study was to investigate the growth performances of mulberry transplants produced in indoor cultivation with modified micro-environments surrounding the root and aerial zones. Small single-node cuttings were used in this study in order to reduce the wastage of softwood stem cuttings in conventional mulberry propagation and were grown in different culture systems to produce a uniformity of mulberry mini-transplants. A combination of culture system and controllable artificial environments aimed to establish an alternative plant-factory process of mulberry transplantation that has potential to be applied to the industrial production of bioactive components from mulberry stock plants.

## 2. Results and Discussion

### 2.1. Effect of Substrate Culture on the Quality of Mulberry Transplants

Single-node cutting of mulberry was subjected to different substrate-assisting systems. Morphological growths of single-node-derived transplants of mulberry were observed after 30 days (Table 1). The survival percentage, fresh weight, root length, and number of branches, leaves, and roots of mulberry single-node cuttings were examined after 30 days of treatment with different substrate cultures. Survival percentage was highest in the soil-based and vermiculite-based systems, especially in the MLL cultivar (96%), while it was lowest in the DRFT hydroponic system (50–65%). Mulberry transplants showed a high percentage of root production in substrates that allow oxygen to dissolve such as in soil- and vermiculite-based cultures but not in the DRFT system, indicating that root zone environment is an important factor for transplant production. Respiration takes place in all viable vegetative tissue. Although single-node cuttings have no leaf, respiration still takes place at a relatively low rate and oxygen is required. Roots of cuttings are sensitive to oxygen deficiency and rot if the medium has insufficient oxygen to complete the respiration process [23]. Consequently, well-aerated substrates such as vermiculite should be considered as oxygen suppliers and key factors influencing plant survival during cutting propagation. Several studies on cutting propagations of many hardwood species reported the benefits of vermiculite for rooting and healthy root formation due to more water-holding capacity and gradual release of nutrients from the vermiculite medium [24,25]. DRFT is a simple hydroponic system that has the advantage of low electrical energy consumption but provides insufficient aeration for the root system [26]. Therefore, choosing an appropriate rooting medium and an effective system is a significant key factor in cutting propagation to achieve optimum rooting in the shortest time.

**Figure 1:**
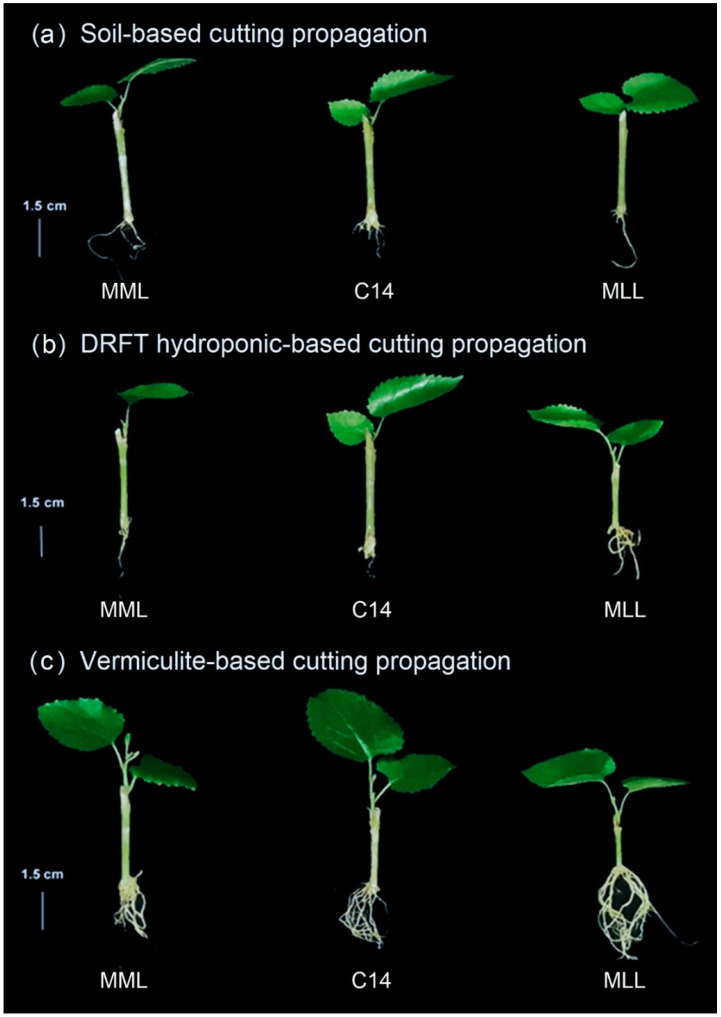
The shoot/root formation of mulberry transplants induced from single–node cuttings of three mulberry cultivars: Myanmar medium leaf (MML), C14, and Myanmar large leaf (MLL), after 30 days of cutting in three different substrates of soil (**a**), DRFT hydroponic– (**b**) and vermiculite–based systems (**c**).

Root length and root number were significantly affected by both culture system and cultivar, as shown in Figure 1. Maximum root length (3.4 cm) and root number (8.4 roots/explant) were obtained from MLL grown in the vermiculite-based system. Several reports indicated that cutting length, node position, and leaf area were important factors affecting root growth. In *M. alba*, cutting length impacted successful rooting but there was no relationship between root diameter and rooting ability [27,28]. By contrast, no significant differences in leaf number were shown by substrate systems and cultivars. This might relate to the low rate of initial leaf emergence, which is a result of root system limitations and temperature effects [29]. Lok et al. [30] noted a significant improvement in multiplication rate in vermiculite-based systems as promising stock plant production under a controlled environment. A wide range of materials can be used as substrates to support single-node cutting propagation including rockwool, perlite, vermiculite, and peat in soilless cultivation systems [31]. To support rapid root growth and attain high productivity, qualities of a substrate should be fine, uniform, well-aerated, and loose [32].

**Figure 2:**
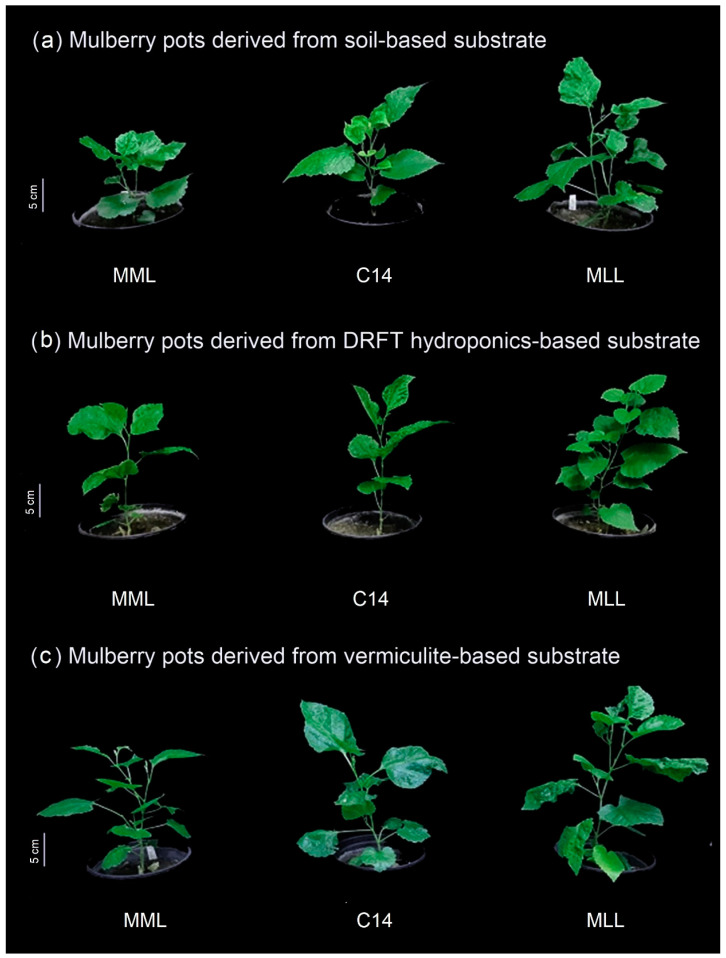
Morphological growth of mulberry cultivars: Myanmar medium leaf (MML), C14, and Myanmar large leaf (MLL) plants, grown in soil-containing pots after transfer from different production systems as soil (**a**), DRFT hydroponic (**b**), or vermiculite (**c**) from induction trays to pots for 60 days.

**Figure 3:**
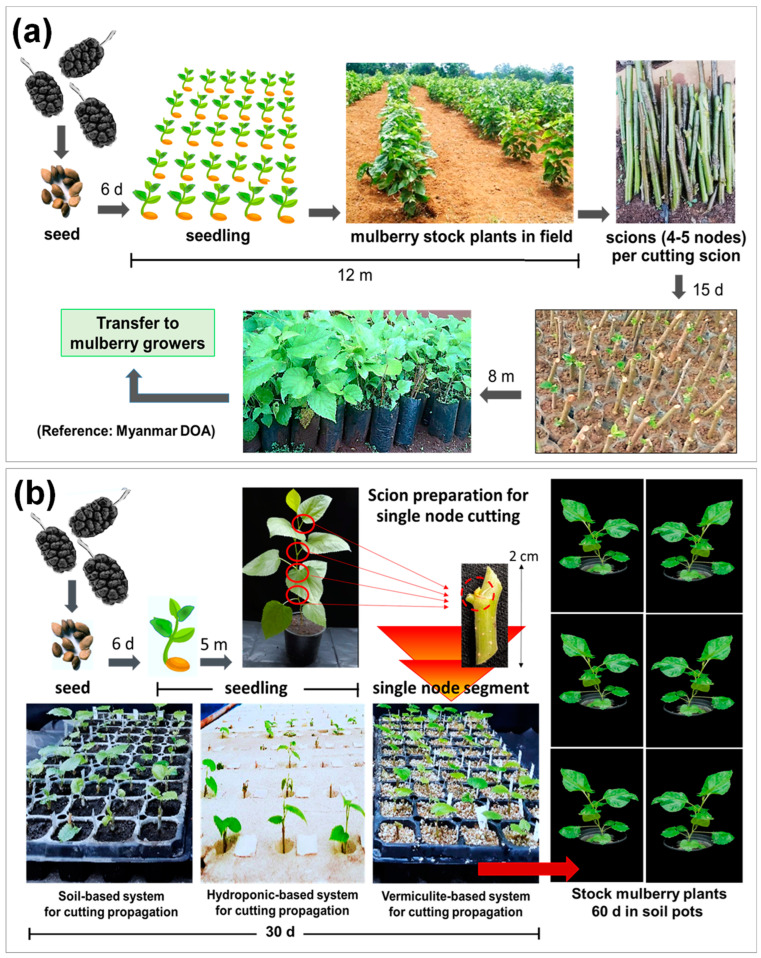
Schematic illustration of mulberry propagation compared between mulberry propagation using softwood stem cutting conventionally suggested by Myanmar DOA (**a**) and the transplants derived from single–node cutting propagation established in this study (**b**).

We continued to monitor the morphological growth of mulberry cultivars in soil-containing pots. All the cutting propagules grew well after 60 days of transplantation (Figure 2). Obviously, the propagules with better root growth obtained from the vermiculite-based system showed the best growth performance, indicating that root production is important for the efficient cutting propagation of *M. alba.* According to a conventional mulberry propagation by Myanmar DOA, the cultivation process required at least 1.5 years from seed to stock plants (Figure 3). In our recent studies, the transplant propagation derived from single-node cutting required about 8 months to obtain the same size of mulberry plants. Moreover, our process provided more propagules than the conventional procedure using softwood stem cutting, especially those derived from the vermiculate-based substrate (V-system). Thus, our study suggested an alternative system for mulberry production, which is more effective in root production, produces mulberry transplants with uniformity, and requires less time for cultivation. This not only overcomes the limitation of propagating season and production time but also facilitates the industrial handling system and distribution of mulberry transplants.

In addition to silkworm feeding, mulberry leaves are widely used in traditional medicine, food, and beverage industries in many countries. Young leaves or tender shoots of mulberry have been consumed as vegetables in some areas of China. It was reported that tender shoots contain bioactive components such as ascorbic acid, carotenoid, total soluble protein, total soluble sugar, fructose, and sucrose, depending on a variety of mulberry [33]. As previously stated, we successfully modified the root zone environment by selecting the most suitable supporting material for mulberry cultivation. The impacts of aerial environments were also investigated. Light signal, which is widely known for its effect on regulating the growth and development of plants, was chosen as a key parameter for the aerial environment. The influences of light signals on mulberry growth and bioactive compound production were further examined.

### 2.2. Growth Performance of Stock Plants under Different Light Intensities

Five-month-old mulberry plants were exposed to three light intensities for 7 days. The shoot length of the MLL cultivar significantly increased when exposed to a light intensity of 200 µmol·m^−2^·s^−1^, while a short-term increase in light intensity did not affect shoot length in the C14 cultivar (Table 2). The root length of mulberry stock plants was not influenced by 7 days of exposure to artificial light. Among all the permutations, MLL showed a higher root length (25.0 cm) at 200 µmol·m^−2^·s^−1^ followed by MML (24.7 cm) at 200 µmol·m^−2^·s^−1^ and MLL (24.5 cm) at 100 µmol·m^−2^·s^−1^ of light intensity. Exposing the plants to 200 µmol·m^−2^·s^−1^ of light intensity for 7 days favored the development of a higher numbers of leaves, especially in MLL and MML cultivars. Regardless of the cultivar, fresh weight gradually increased with an increase in light intensity.

Our results demonstrated that the optimal light intensity for growing mulberry transplants with vigorous growth, higher fresh weight, and leaf biomass was 200 µmol·m^−2^·s^−1^. However, the responses varied among mulberry cultivars as the genotypic effect plays a major role in responses to different light quantities. MML demonstrates its superior potential for use as a candidate cultivar for indoor production for both transplant quality and growth performances. As mulberry contains several useful compounds that can be used as functional ingredients for both food and medical purposes, we further investigate whether light spectral qualities affect bioactive compound production in indoor cultivation.

### 2.3. Effect of LED Light Spectra in a Semi-Closed Indoor System on Production of Functional Ingredients in Mulberry Leaves

The mulberry transplants were grown indoors under white light before exposing to different light spectra at a light intensity of 200 µmol·m^−2^·s^−1^ for 7 days. The effects of different artificial light spectra and their interaction with cultivars on the nutritional quality of mulberry plants were investigated. The results demonstrated significant effects of different light spectra on the accumulation of total soluble proteins, total soluble sugar, and total phenolic content (Table 3). Among the different cultivars, MML leaves exposed to the white-light spectrum accumulated higher total soluble proteins (12.0 ± 1.8 mg·g^−1^ FW) than the other cultivars and light spectra analyzed. Nevertheless, the leaves from C14 exposed to the blue light spectrum accumulated the least total soluble protein (6.2 ± 1.9 mg·g^−1^ FW), approximately half of the highest content obtained. A similar result was observed in *P. vulgaris* L., with the highest total protein content detected under white fluorescent light compared to other light spectra [34].

Similar to total soluble proteins, accumulation of soluble sugars in MLL was highest under white light (65.1 ± 8.5 mg·g^−1^ DW). All tested cultivars accumulated a significantly higher soluble sugar content when the plants were exposed to white light than the blue or red light (Table 3). These findings are consistent with those of Cui et al. [35], who found that white light treatment markedly enhanced the soluble sugar content of ‘Yanghua’ radish sprouts. Though the highest content of total soluble sugar was noted in the leaves of mulberry treated with white light and the lowest was noted in those treated with red and blue light, neither cultivars nor their interaction with different light spectra showed a statistical influence on total soluble sugar content.

By contrast, a statistically significant effect of light spectra, cultivars, and the interaction between light spectra and cultivars was observed on the total production of phenolic compounds. The accumulation depended on the specific responses of each cultivar to the light spectrum. A higher content of phenolic compounds in MLL was promoted by blue light, whereas the preferable light spectra of MML and C14 were white and red spectra, respectively (Table 3). Among all the permutations and combinations analyzed, a higher phenolic content (1339 ± 75.9 µg·g^−1^ FW) was accumulated in MLL plants under the blue-light spectrum, while the least phenolic content (379.5 ± 51.2 µg·g^−1^ FW) was observed in MML under the red-light spectrum. The results showed that the total phenolic content in all cultivars was noticeably affected by different light spectra and mulberry cultivars.

In addition, metabolic profiles of ethanol extracts from leaves of three mulberry cultivars showed excellent bioactive compound production under the artificial environment, with several bioactive compounds such as terpene, furan and derivatives, fatty acid and conjugates, and other hydrocarbons (Table 4). Several bioactive compounds in mulberry leaves have also shown protective and therapeutic effects such as anticancer, anti-inflammatory, anti-obesity, antiaging, anti-microbial, and anti-diabetic properties, and may also ameliorate chronic cardiovascular and neurodegenerative diseases [35,36,37,38].

Among the detected terpenoid compounds, neophytadiene and phytol were highly expressed in this study. Neophytadiene was previously reported for antidiabetic, antioxidant, antibacterial, and antimalarial activities [39,40]. The total content of this compound was recorded as highest (82.4%) in MLL plants exposed to white-spectrum light, while C14 produced twofold less than the highest amount in the red-light condition. The C14 accumulated more neophytadiene in white and blue light (52.8–55.9%), as well as the highest level of this compound in MML under white-light treatment, indicating that all tested cultivars showed positive responses to white-spectrum light in promoting neophytadiene accumulation. Diterpene phytol present in the extract of mulberry leaves possessed antidiabetic, antioxidant, and antibacterial properties [39]. Under white-spectrum light, the leaves of C14 had the highest total content (93.7%). Similarly, the highest phytol content (91.4%) was also detected in leaves of MML cultivated in the same light spectrum. Under red-light treatment, the leaves of MLL accumulated an 88.8% relative content of phytol. Conversely, the other cultivars produced the least amount under this spectrum.

Fatty acid and conjugates play very important roles in metabolism that support storage and energy transportation. They were reported to possess several bioactivities including antioxidant and antibacterial activities [36,41,42,43,44]. As one of the most abundant fatty acids reported in this study, palmitic acid is widely known as an antioxidant with antibacterial, antifungal, anti-inflammatory, and hypocholesterolemic effects [41,42]. Although the metabolic profile revealed that palmitic acid was highly abundant in all cultivars of mulberry, variations in content were observed among the mulberry cultivars and light treatments. The highest level was detected in MLL exposed to the red light with a relative content of 133.5%, while the leaves of C14 showed the highest level (108.6%) under blue-spectrum light. High levels of palmitic acid were also noted in MLL (91.3%) and MML (120.7%) exposed to white-spectrum light. Another abundant fatty acid conjugate is linolenic acid methyl ester that possesses antioxidant activity and protects against cardiovascular diseases, neuronal degeneration, and aging skin cancer [36,44]. All cultivars produced high levels of linolenic acid methyl ester in white and blue spectra. The highest total content was observed in C14 exposed to blue spectra. In the presence of red light, variations in linolenic acid methyl ester were clearly observed. The highest concentration (86.4%) was found in leaves of MLL. MML leaves, however, had the lowest content (45.2%).

**Figure 4:**
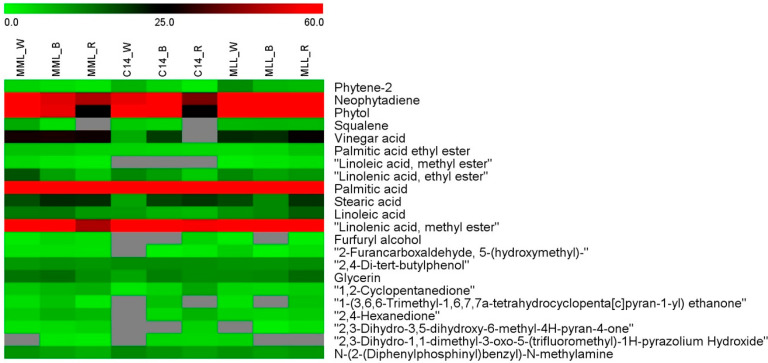
Heatmap demonstrated variations in metabolic profiles detected in leaves of three *M. alba* L. cultivars after exposure to different light spectra for 7 days. Relative abundance of the compounds was represented as a relative content (%). Green color indicates low amounts of compounds, whereas those with high amounts are colored red.

A heatmap analysis revealed differential levels of volatile metabolites detected in leaves of three mulberry cultivars after exposure to different light conditions (Figure 4). The heatmap confirmed the abundances of neophytadiene, phytol, palmitic acid, and linolenic acid methyl ester, as shown in red color. The amounts of these compounds were abundant across all analyzed light spectra in MLL, indicating that this cultivar showed positive responses to a wide range of light wavelengths. Nevertheless, MML and C14 produced higher contents under white and blue spectra but had lower levels when exposed to red-spectrum light. A similar result was previously observed in *A. annua* as a specific wavelength of 445 nm present in both white and blue spectra induced a greater abundance of artemisinin, artemisinic acid, and some useful terpenoid compounds [40]. Variations in biochemical responses and their interaction with the light spectrum and mulberry cultivar suggested that bioactive compounds in mulberry respond to light quality in a cultivar-dependent manner.

Our result demonstrated that plant factories with artificial lighting combined with hydroponics or substrate (soilless) cultivations can be applied to overcome the limitations of climatic variables, low space, or lack of stock plants. This alternative growing method with high resource use efficiency (RUE) has been used to produce adequate yields of quality transplants [45,46]. In addition, supplementing appropriate light conditions provided a promising approach to enhance bioactive compounds and nutraceuticals in mulberry. Nevertheless, metabolite productions were also influenced by mulberry cultivars. As LED spectra had no significant effect on metabolite production in MLL, field cultivation may be considered to grow this cultivar. Interestingly, MML and C14 exhibited diverse biochemical responses when exposed to different light spectra. Therefore, MML and C14 have the potential to be used as plant materials to assess the effect of light on nutritional quality. These will provide an alternative system to produce active ingredients for pharmaceutical and functional food.

## 3. Material and Methods

### 3.1. Preparation of Plant Materials

Three mulberry cultivars of Myanmar large leaf (MLL), Myanmar medium leaf (MML), and C14 were obtained from the Sericulture Research and Development Center, Pyin-Oo-Lwin, Mandalay Region, Myanmar. Seeds were sown in a soil-based system and grown for 5 months to produce uniform and healthy stock plants. In this study, mulberry propagation was carried out using single-node cutting, instead of softwood stem cutting used in conventional mulberry propagation (Myanmar DOA). Single-node cutting materials of mulberry were excised from the 5-month-old stock plants and then plugged into different substrate-assisting systems. After one month, survival percentage; shoot height (cm); number of branches, and leaves, and roots; root length (cm); fresh weight (g) per cutting plant were recorded.

### 3.2. Substrate Culture System for Transplant Production Derived from Single-Node Cutting

The excised single-node segments of mulberry were cultivated in trays using three different substrates including commercial-soil-based and two soilless systems using vermiculite-based and hydroponic systems. A commercial soil mixture (EC = 2.687 dSm^−1^; pH = 5.5; organic matter = 90.36%; total nitrogen = 0.17%; total phosphorus = 0.07%; total potassium = 1.19%) in 72-well plug trays (21¼-inches long, 11¼-inches wide, and 2¼-inches high) was used as the soil-based system. The vermiculite-based system used clean medium-grain vermiculite as the supporting material in similar-sized plug trays. The hydroponic system (Hydro-set^®^, Hygreen Hydroponics Farm, Thailand) employed a dynamic root floating technique (DRFT) supported by a commercial sponge as the supporting material. Experiments were performed by incubating single-node materials of three substrate treatments on the culture shelves in a semi-closed indoor system under 25 ± 2 °C and 65 ± 2%RH. The cutting treatments were illuminated with LED light (445 and 554 nm) at a light intensity of 150 µmol·m^−2^·s^−1^ and 16 h photoperiod. Fresh nutrient solution was changed at 7-day intervals for the growing period of 30 days. All treatments were supplemented with Hoagland nutrient solution at an adjusted pH of 5.8–6.

### 3.3. Mulberry Culturing System for Light Treatments

Five-month-old stock plants grown from single-node transplants, each with 5–7 nodes, were incubated under white LED light in a semi-closed indoor system for 30 days, and then incubated under dark conditions for 24 h before being subjected to short-period light exposure treatment. The transplants were illuminated with varying light intensities and different spectral compositions of LED lighting. For treatment of light intensity, the growth performances of mulberry stock plants were examined after illumination with different light intensities for 7 days at 50, 100, or 200 µmol·m^−2^·s^−1^ of white LED light (445 and 554 nm) with a 16 h photoperiod. On the other hand, light spectra that affected transplant quality were investigated. The uniform stock plants were treated with various LED spectra: blue (445 nm), white (445 and 554 nm), or red (660 nm), for 7 days. All experiments were conducted in a semi-closed indoor system with artificial light at the Faculty of Science, Mahidol University, Bangkok, Thailand (location: 13°45′51.8″ N 100°31′28.6″ E).

### 3.4. Determination of Biochemical Compounds and Metabolic Profiles

Fresh leaves of mulberry (0.1 g) were ground into powder using liquid nitrogen. The powder was suspended in 2 mL of absolute ethanol and sonicated for 20 min at 25 °C. After centrifuge, the supernatant was filtered through a 0.45 µm filter membrane (Millipore, Burlington, MA, USA) and maintained at −20 °C until analysis. Gas Chromatography–Mass Spectrometry (GC–MS) was performed to identify and quantify the volatile metabolites. Aliquots of 250 µL of the extract solutions were injected in a GC–MS Agilent model 6890N (Agilent Technologies Inc., Santa Clara, CA, USA) using an HP-Innowax column (30 m × 0.25 mm, film thickness 0.25 µm) with splitless mode. The data were searched against the WILEY No.7 database and only compounds with more than 80% similarity were used for data analysis. Relative abundance of the compounds was quantified by comparing their peak areas with the internal standard (methyl heptadecanoate, C17) and represented as a relative content (%).

### 3.5. Extraction and Determination of Total Soluble Protein

Total protein content was analyzed by the Bradford method [47]. A fresh leaf sample (0.1 g) was blended in a mortar with liquid nitrogen and 1 mL of 0.2 M phosphate-buffered solution (pH 7.0) was added. The extract was centrifuged at 13,000 rpm for 15 min at 4 °C. An aliquot of 0.1 mL of supernatant was then added with 5 mL of Coomassie brilliant blue G-250 solution (25% *v*/*v*). The supernatant changed to a blue color after 2 min and the absorbance was read using a UV–Vis Spectrophotometer (GENESYS™, Thermo Fisher Scientific, Waltham, MA, USA) at a wavelength of 595 nm.

### 3.6. Extraction and Determination of Total Soluble Sugar (TSS)

The total sugar content (TSS) was determined using the phenol sulfuric acid method [48]. A sample of dried leaves (0.01 g) was ground in liquid nitrogen using a mortar and then 1 mL of distilled water was added. The extract was placed in a water bath at 85 °C for 30 min. Next, 1 mL of the 5% phenol solution and 5 mL of 98% sulfuric acid were added. The absorbance of the supernatant was measured at 490 nm using a GENESYS 10S UV–Vis Spectrophotometer, and the result was represented in milligrams per gram of dry leaf weight (mg·g^−1^ DW). Dilutions of D-glucose solution were prepared and used to generate a standard curve for total sugar determination.

### 3.7. Extraction and Determination of Total Phenolic Content

A Folin–Ciocalteu colorimetric method was used to evaluate the total phenolic content in mulberry leaves [49]. A fresh leaf sample (0.1 g) was ground in a mortar with liquid nitrogen and 1 mL of 60% methanol (*v*/*v*) was added. The extract (0.5 mL) was then diluted with 3.5 mL of distilled water. After dilution, 0.25 mL of 2N Folin’s reagent was added to the solution, followed by 0.75 mL of 20% (*w*/*v*) sodium carbonate. The resulting solution was mixed thoroughly and maintained at room temperature for 15 min to complete the reaction under dark conditions. The reaction was carried out in three replications, each of which was repeated in triplicate and then averaged. The absorbance was measured at 760 nm using a GENESYS 10S UV–Vis Spectrophotometer. Using gallic acid as a standard, the total phenolic content in the sample was calculated and expressed as micrograms of gallic acid equivalent per gram of fresh leaf weight (µg GAE·g^−1^ FW).

### 3.8. Statistical Analysis

The experiment of single-node cutting (as defined in 2.2) was designed as a 3 × 3 completely randomized design (CRD) with 50 replications per treatment (1 node = 1 replication), while the experiments of light intensity and light spectrum (as defined in 2.3) were performed using four biological replications (1 pot = 1 replication) and three experimental replications per treatment. Data were analyzed using PASW Statistics 18.0.0. Results were expressed as mean (±SD). One-way ANOVA, followed by Duncan’s Multiple Range test (DMRT), was used to determine the statistical differences among the treatments. In the case of missing data occurring, statistical analyses were performed using one-way ANOVA followed by Turkey’s post hoc test.

## 4. Conclusions

Even though stem cuttings, leaf cuttings, root cuttings, and single-node cuttings are widely used to propagate a wide range of perennials, deciduous shrubs, and some woody plants, the mulberry propagation in Myanmar generally uses softwood stem cuttings. The mulberry plants are harvested intensively under the tropical climate, resulting in exhaustion and renewed plantation requirement. This present research demonstrated an alternative conceptualization of mulberry transplants production from single-node cutting. Two major advantages might be exemplified. First, our established system consisted of mulberry transplants derived from single-node cuttings grown in a vermiculite-based substrate, supplied with adequate and proper nutrient solution. This applied system generated qualified mulberry transplants with uniformity and vigorous growth, free from latent contamination of diseases or insects. The propagating period using the V-based substrate (90 days) was shorter than those produced by conventional stem cutting (more than 8 months). It is also suggested that the mulberry transplants established in this present study potentially facilitate the industrial production of clean stock plants and logistic handling systems. These stock plants can be efficiently reprocessed and can overcome the previous limitation of production space, season, labor, etc.

In addition to its advantage of propagation efficiency, these indoor transplants can be utilized as sources of useful compounds. Although mulberry leaf tea is used as a source of an excellently refreshing and nourishing drink due to its natural taste, aroma, and color, the additional benefits of active ingredients found especially in mulberry are attractive for health products. In this study, we demonstrated a new concept to use mulberry leaves as alternative sources of functional ingredients for humans, not just for herb tea. High-quality mulberry leaves produced indoors can support industrial production with good manufacturing practice (GMP) to serve the commercial demands for edible fiber, and nutraceutical compounds in food, feed, and medicinal industries. The properties of mulberry leaves in terms of biomass and the production of bioactive compounds can be modified using physical elicitors such as light intensity and wavelength. In our study, light intensity and short-period treatment of specific LED light spectra applied to the aerial zone of mulberry transplants could significantly induce different nutritional and nutraceutical compounds produced in leaves. This might open the opportunity to precisely design the raw material properties to meet the requirements of consumers. However, the mulberry cultivar plays a key important role in the variated responses to the PFAL system and light elicitation. According to the overall parameters gained from our study, we suggested that the MLL cultivar performed as an elite candidate when compared to MML and C14 cultivars. The system established in this study can enable large-scale and year-round production of qualified raw materials. It is also suitable for up-scaled industrialization under controllable or modified conditions that support the precision production of each specific purpose.

## Figures and Tables

**Table 1:** Survival percentage, fresh weight, branch, leaf and root number, and root length of mulberry single–node cuttings from three cultivars: Myanmar medium leaf (MML), C14, and Myanmar large leaf (MLL), 30 days after insertion of single–node segments to three different systems of soil, DRFT hydroponic, or vermiculite–based substrates supplemented with Hoagland solution.

Substrate (s) Cultivar (cv)	Survival (%)	Branch No.	Leaf No.	Root No.	Root Length (cm)
Soil					
MML	83	1.2 ± 0.4 ^b^	2.4 ± 1.0	4.4 ± 0.9 ^c^	2.1 ± 0.9 ^bc^
cv C14	83	1.3 ± 0.5 ^ab^	1.8 ± 0.4	4.6 ± 0.8 ^c^	0.3 ± 0.1 ^d^
cv MLL	96	1.3 ± 0.5 ^ab^	2.4 ± 0.6	5.2 ± 0.9 ^c^	1.6 ± 0.7 ^c^
DRFT Hydroponic					
cv MML	56	1.4 ± 0.5 ^ab^	2.5 ± 1.2	4.9 ± 1.0 ^c^	2.2 ± 0.8 ^bc^
cv C14	61	1.1 ± 0.4 ^b^	2.3 ± 0.5	5.1 ± 1.0 ^c^	0.3 ± 0.1 ^d^
cv MLL	65	1.4 ± 0.5 ^ab^	2.5 ± 1.2	6.3 ± 1.0 ^b^	2.6 ± 1.0 ^ab^
Vermiculite					
cv MML	87	1.2 ± 0.4 ^b^	2.0 ± 0.5	5.3 ± 1.0 ^c^	2.4 ± 1.0 ^bc^
cv C14	87	1.8 ± 0.6 ^a^	2.1 ± 0.6	6.5 ± 1.0 ^b^	1.8 ± 1.0 ^bc^
cv MLL	96	1.4 ± 0.5 ^ab^	2.3 ± 0.8	8.4 ± 1.0 ^a^	3.4 ± 0.9 ^a^
Substrate (s)		*	ns	**	**
Cultivar (cv)		ns	ns	**	**
s × cv		**	ns	**	**

Different letters within columns indicate significant differences (* at *p* ≤ 0.05) or highly significant differences (** at *p* ≤ 0.01) of means ± S.D. and analyzed by Tukey’s test; ns = non-significant difference.

**Table 2:** Effect of different light intensities and cultivars on shoot length, branch number, leaf number, root length, and fresh weight of mulberry plants exposed to artificial light for seven days.

Light Intensity (µmol·m^−2^·s^−1^)	Cultivar (cv)	Fresh Weight (g)	Shoot Height (cm)	Root Length (cm)	Branch No.	Leaf No.
50	MML	12.2 ± 2.1	27.6 ± 2.2	21.2 ± 1.2	3.3 ± 0.6	14.3 ± 3.1
50	C14	12.4 ± 0.6	24.1 ± 1.9	17.8 ± 0.8	2.3 ± 0.6	8.7 ± 2.1
50	MLL	13.3 ± 2.5	27.4 ± 1.4	20.2 ± 2.5	3.7 ± 0.6	13.7 ± 1.2
100	MML	12.1 ± 2.1	29.0 ± 1.7	23.0 ± 2.0	3.0 ± 0.0	13.7 ± 3.1
100	C14	13.6 ± 0.2	25.8 ± 2.8	20.1 ± 4.3	2.7 ± 0.6	12.0 ± 1.0
100	MLL	15.2 ± 2.7	29.0 ± 1.7	24.5 ± 4.2	3.3 ± 0.6	13.0 ± 2.0
200	MML	15.0 ± 0.8	27.7 ± 2.1	24.7 ± 4.5	3.0 ± 0.0	15.0 ± 2.6
200	C14	13.9 ± 2.4	26.1 ± 0.8	19.7 ± 5.0	3.3 ± 0.6	13.0 ± 3.6
200	MLL	17.1 ± 2.0	29.7 ± 1.2	25.0 ± 2.6	3.7 ± 0.6	15.7 ± 3.8
Light intensity (L)		*	ns	ns	ns	ns
50		12.6 ^b^	26.4	19.8	3.1	12.2
100		13.6 ^ab^	28.0	22.5	3.0	12.9
200		15.3 ^a^	27.8	23.1	3.3	14.6
Cultivar (cv)		ns	**	*	*	*
MML		13.1	28.1 ^a^	23.0 ^a^	3.1 ^ab^	14.3 ^a^
C14		13.3	25.4 ^b^	19.2 ^b^	2.8 ^b^	11.2 ^b^
MLL		15.2	28.7 ^a^	23.2 ^a^	3.6 ^a^	14.1 ^a^
L × cv		ns	ns	ns	ns	ns

The different letters within the column show a significant difference (*) at *p* ≤ 0.05, or a highly significant difference (**) at *p* ≤ 0.01 of means ± S.D. analyzed by Duncan’s Multiple Range test (DMRT) using four biological replications and three experimental replications per treatment; ns = non-significant difference.

**Table 3:** Total contents of protein, soluble sugars, and phenolic compounds detected in leaves of mulberry plants that were exposed to different light spectra of white (445, 554 nm), blue (445 nm), or red (660 nm) for 7 days.

Spectra (s)	Cultivars (cv)	Total Protein Content (mg·g^−1^ FW)	Total Soluble Sugar Content (mg·g^−1^ DW)	Total Phenolic Content (µg·g^−1^ FW)
White (445, 554 nm)	MML	12.0 ± 1.8 ^a^	64.7 ± 6.8 ^a^	589.3 ± 87.5 ^d^
	C14	9.8 ± 2.2 ^abc^	47.8 ± 3.3 ^a^	520.9 ± 92.8 ^de^
	MLL	10.4 ± 1.6 ^abc^	65.1 ± 8.5 ^a^	1057.5 ± 154.9 ^b^
Blue (445 nm)	MML	6.2 ± 1.9 ^c^	22.2 ± 3.7 ^b^	513.4 ± 36.8 ^de^
	C14	9.7 ± 1.5 ^abc^	27.0 ± 9.9 ^b^	531.3 ± 67.4 ^de^
	MLL	8.9 ± 0.5 ^abc^	27.7 ± 4.6 ^b^	1339.3 ± 75.9 ^a^
Red (660 nm)	MML	7.3 ± 1.4 ^bc^	23.7 ± 10.8 ^b^	379.5 ± 51.2 ^e^
	C14	10.5 ± 3.1 ^ab^	22.8 ± 11.7 ^b^	575.9 ± 80.4 ^d^
	MLL	11.0 ± 2.4 ^ab^	25.4 ± 14.4 ^b^	781.2 ± 67.4 ^c^
Spectra (s)		*	**	**
Cultivars (cv)		ns	ns	**
s × cv		*	ns	**

Three mulberry cultivars MML, C14, and MLL were exposed to different light spectra using LED light at 200 µmol·m^−2^·s^−1^ for a 16 h photoperiod and incubated under 25 °C, 70 ± 5%RH for 7 days. The different letters within the column show a significant difference (*) at *p* ≤ 0.05, or a highly significant difference (**) at *p* ≤0.01 of means ± S.D. analyzed by Duncan’s Multiple Range test (DMRT) with four biological replications and three experimental replications per treatment; ns = non-significant difference.

**Table 4:** Metabolic profiles of mulberry leaf extract analyzed by GC–MS. Three cultivars of mulberry (MML, C14, and MLL) were exposed to different light spectra.

Compound	Compound Name	W (445, 554 nm)			B (554 nm)			R (660 nm)		
		MML	C 14	MLL	MML	C 14	MLL	MML	C 14	MLL
Terpene	Phytene-2	4.3	7.5	11.3	3.5	4.4	7.2	2.6	2.1	6.7
	Neophytadiene	65.8	52.8	82.4	52	55.9	62.3	45.5	39.2	71.8
	Phytol	91.4	93.7	67.6	52.6	57.3	61.3	25.2	25.7	88.8
	Squalene	8.4	5.2	7.0	3.5	4.4	7.3	-	-	6.9
Fatty acid and conjugates	Vinegar acid	28.4	8.3	20.5	28.9	18.8	20.2	28	-	26.4
	Palmitic acid ethyl ester	5.9	4.4	3.6	5.6	4.1	3.9	4.4	3.7	5.9
	Linoleic acid, methyl ester	3.9	-	1.9	2.6	-	2.5	2	-	3.5
	Linolenic acid, ethyl ester	16.6	11.7	11.5	9.4	9.6	9	5.8	5.2	10.2
	Palmitic acid	120.7	77.1	91.3	111.5	108.6	81	95.2	86.1	133.5
	Stearic acid	17.2	9.1	17.6	20.5	18.1	11.5	20.2	19.5	19.9
	Linoleic acid	13.1	9.8	10.4	13.2	7.6	11.5	10	6.9	15.7
	Linolenic acid, methyl ester	73.7	80.9	77.8	89.9	96.3	74.5	45.2	54.7	86.4
Furan and derivatives	Furfuryl alcohol	2.1	-	1.9	4.1	-	-	3.3	4.5	1.6
	2-Furancarboxaldehyde, 5-(hydroxymethyl)-	2.9	-	4.9	3.3	1.6	1.2	3	2.3	3.3
Phenolic compound	2,4-Di-tert-butylphenol	10	11	10.5	10.8	11.4	10.3	10.2	11.2	11.2
	Glycerin	13.7	8.9	11.4	14.4	12.5	11.6	11.1	11.2	14.5
Other hydrocarbons	1,2-Cyclopentanedione	5.3	2.4	4.3	7.2	4.5	3.9	7.9	8.9	5
	1-(3,6,6-Trimethyl-1,6,7,7a-tetrahydrocyclopenta[c]pyran-1-yl) ethanone	3.5	-	2.9	6.1	6	-	2.3	-	5.9
	2,4-Hexanedione	5.1	-	4.6	7.7	4	4.1	6.8	7.4	5.1
	2,3-Dihydro-3,5-dihydroxy-6-methyl-4H-pyran-4-one	2.9	-	-	3.6	-	1.7	4.5	4.4	3.1
	2,3-Dihydro-1,1-dimethyl-3-oxo-5-(trifluoromethyl)-1H-pyrazolium Hydroxide	-	-	4.3	2.4	4.1	-	2.1	3.9	-
	N-(2-(Diphenylphosphinyl)benzyl)-N-methylamine	10.6	11.8	10.5	9.1	10.3	9.5	7.9	9.4	10.1

Relative abundance of the compounds was quantified by comparing their peak areas with the internal standard (methyl heptadecanoate, C17) and represented as a relative content (%). Only the compounds with more than 80% similarity quality to the Wiley No. 7 database are shown.

## Data Availability

Not applicable.
